# LB1528. Results from ACTIV-6: A Decentralized, Double-Blinded, Randomized, Placebo-Controlled Platform Trial of Repurposed Drugs for the Treatment of Mild-to-Moderate COVID-19

**DOI:** 10.1093/ofid/ofac492.1874

**Published:** 2022-12-15

**Authors:** Matthew W McCarthy

**Affiliations:** Weill Cornell Medicine, New York, NY

## Abstract

**Background:**

The effectiveness of fluvoxamine to shorten symptom duration or prevent hospitalization among outpatients with mild-to-moderate coronavirus 2019 (COVID-19) is unclear. Tolerability has also been identified as a potential limiting factor for 100 mg twice daily of fluvoxamine. We evaluated the efficacy of low-dose fluvoxamine 50 mg twice daily for 10 days compared with placebo for the treatment of early mild-to-moderate COVID-19.

**Methods:**

ACTIV-6 is an ongoing, decentralized, double-blind, randomized, placebo-controlled platform trial testing repurposed medications in outpatients with confirmed SARS-CoV-2 infection. Non-hospitalized adults aged ≥ 30 years, experiencing ≥ 2 symptoms of acute infection for ≤ 7 days were randomized to fluvoxamine 50 mg twice daily for 10 days or placebo. The primary outcome was time to sustained recovery, defined as the third of 3 consecutive days without symptoms. Secondary outcomes included composites of hospitalization or death with or without urgent care or emergency department visit by day 28.

**Results:**

Of those eligible for the fluvoxamine arm, 675 were randomized to and received fluvoxamine; 619 received concurrent placebo. Sixty-six percent of the study population reported at least 2 doses of a COVID-19 vaccine. There was no evidence of improvement in time to recovery with fluvoxamine compared with placebo (hazard ratio [HR] 0.96, 95% credible interval [CrI] 0.86–1.06; posterior probability for benefit [HR > 1]=0.2). Sixteen participants (2.4%) in the fluvoxamine arm had urgent care or emergency department visits or were hospitalized compared with 11 (1.8%) in the pooled, concurrent placebo arm (HR 1.5, 95% CrI 0.5–3.0; posterior probability for benefit [HR < 1]=0.1725). No participant in either arm was hospitalized, and no deaths occurred. Adverse events were uncommon in both arms.

**Conclusion:**

Treatment with low-dose fluvoxamine 50 mg dosed twice daily for 10 days did not result in improved time to recovery among outpatients with COVID-19 in the United States during the delta and omicron variant surges.

**Disclosures:**

**All Authors**: No reported disclosures.

